# Mechanical Pain is a Main Type of Pain in Patients With Advanced Knee Osteoarthritis

**DOI:** 10.1155/prm/8356050

**Published:** 2025-11-24

**Authors:** Qiyao Li, Chenchang He, Rui Huang, Xiang Gao, Li Li, Pei Fan

**Affiliations:** ^1^The Second Affiliated Hospital of Wenzhou Medical University, Wenzhou, Zhejiang, China; ^2^Department of Orthopedics, The Second Affiliated Hospital of Wenzhou Medical University, Wenzhou, Zhejiang, China; ^3^Zhejiang Provincial Key Laboratory of Anesthesiology, The Second Affiliated Hospital of Wenzhou Medical University, Wenzhou, Zhejiang, China

**Keywords:** knee ostoarthritis (KOA), mechanical pain, prevalence, risk factors

## Abstract

**Objectives:**

This study aimed to investigate the prevalence and risk factors of mechanical pain in patients with advanced knee osteoarthritis (KOA), providing insights for targeted treatment approaches.

**Methods:**

We conducted a cross-sectional study involving 920 patients with KOA. The sample size was determined using the formula *n*=(*Z*^2^∗*P*∗(1 − *P*))/*E*^2^, assuming a 95% confidence interval (CI) and a 5% margin of error. Data on demographics and affected knee parameters, including age, sex, body mass index (BMI), affected side, Western Ontario and McMaster Universities Osteoarthritis Index (WOMAC) scores, range of motion, degree of varus, and numeric rating scale (NRS) were collected. Pain was categorized using the painDETECT questionnaire and WOMAC scores to differentiate between simple mechanical pain, mixed mechanical pain, and probable neuropathic pain (NP).

**Results:**

Among participants, 43.48% experienced simple mechanical pain, 33.48% had mixed mechanical pain, and 23.04% reported probable NP. Significant differences were observed in the total WOMAC scores, range of motion (bend), and NRS across the three groups. Gender distribution varied significantly, with a higher proportion of female patients in each pain category. Notably, NRS on the affected side was moderately correlated with the total WOMAC pain score (*r* = 0.500, ^∗^*p* < 0.05). Moreover, female patients exhibited significantly higher WOMAC pain scores (6.28) compared with males (6.08), and women with a WOMAC pain score > 4 had an odds ratio (OR) of 2.462 (95% CI: 1.766–3.433, ^∗^*p* < 0.05) compared with those with a score ≤ 4.

**Conclusions:**

Mechanical pain is highly prevalent in patients with advanced KOA. Identifying the specific type of mechanical pain and associated risk factors, such as female gender and higher NRS score, can facilitate personalized pain management.

## 1. Background

Osteoarthritis (OA) is an extremely common joint disease affecting an estimated 240 million people worldwide and is characterized by significant symptoms and limited joint movement [1]. It can affect almost any joint in the body, with knee osteoarthritis (KOA) being particularly common. Its incidence gradually increases with age and is higher in women than in men [2]. According to statistics, the incidence of knee arthritis in adults aged 60 years and older is about 10% in men and as high as 13% in women [3]. KOA not only causes patients to gradually lose joint function, which leads to progressive disability and severely reduces their quality of life but also often leads to depressive symptoms due to persistent chronic pain [4]. Therefore, given the chronic nature of OA, which is characterized primarily by pain, pain management and lifestyle adjustments alone are not enough. For the treatment of KOA pain, we still need to conduct more in-depth exploration and research.

The International Association for the Study of Pain (IASP) divided pain into three categories, including nociceptive pain, neuropathic pain (NP), and nociplastic pain [5, 6], providing an important classification direction for KOA pain from different sources. According to the IASP definition of nociceptive pain, which described as pain attribute to the activation of the peripheral receptive terminals of primary afferent neurons in response to chemical, mechanical, or thermal stimuli [7], mechanical pain was subdivided into pain induced mechanical stimulation [8]. Mechanical pain, a type of nociceptive pain, mainly converts mechanical signals into electrochemical signals through mechanosensitive ion channels (MSICs) and transmits them to the central nervous system [9]. It is generated by harmless and harmful mechanical stimuli detected by sensory neurons of low-threshold and high-threshold mechanoreceptors in the dorsal root ganglion [10]. Most KOA patients initially experience pain when walking or stairclimbing and gradually develop pain when standing, and some also feel pain when sitting or lying down. Thus, pain induced by mechanical load, such as walking, stair climbing, and standing in patients with KOA, is classified as mechanical pain.

Mechanical pain is an important manifestation of KOA pain; however, research on this type of pain is limited, and accurate identification and treatment of mechanical pain remain a challenge. Therefore, through the analysis of clinical data of patients with advanced KOA, this study aims to understand the prevalence rate and possible related risk factors of mechanical pain in patients with advanced KOA, providing data support for targeted treatment of mechanical pain in KOA.

## 2. Material and Method

### 2.1. Moral Statement

The protocol for this analytical cross-sectional study was approved by the Ethics Committees of the Second Affiliated Hospital of Wenzhou Medical University. Written informed consent was obtained from all participating patients.

### 2.2. Inclusion and Exclusion Criteria

Inclusion criteria were (1) patients diagnosed with primary KOA according to American College of Rheumatology criteria; (2) patients with KOA awaiting total knee arthroplasty (TKA); and (3) patients evaluated as having a Grade 4 radiological grade according to the Kellgren–Lawrence (K–L) grading scale (0 = none, 1 = doubtful, 2 = minimal, 3 = moderate, and 4 = severe).

Exclusion criteria were (1) inflammatory arthritis (including rheumatoid arthritis, spinal arthritis, and gouty arthritis); (2) autoimmune diseases (including connective tissue diseases and hemophiliac arthritis); and (3) the presence of pain unrelated to KOA.

### 2.3. Data Collection

The sample size required for this study was calculated using the standard formula for proportion estimation: *n*=(*Z*^2^∗*P*∗(1 − *P*))/*E*^2^. With a 95% confidence interval (CI) (*Z* = 1.96), an assumed proportion (*P*) of 0.65, and a margin of error (E) of 5%, the initial sample size was determined to be 350. To ensure sufficient statistical power for subgroup analyses and accommodate potential missing data, we targeted enrollment of approximately 900 patients. Ultimately, 920 patients (936 knee observations, including 16 bilateral cases) were recruited from our institution between January 3, 2021, and August 10, 2023. Demographics and affected knee parameters of all patients were collected as shown in Table 1, including age, sex, body mass index (BMI), affected knee side, Western Ontario and McMaster Universities Osteoarthritis Index (WOMAC) total score, range of motion, degree of varus, and numeric rating scale (NRS).

Knee function was evaluated using the WOMAC score [11]. This tool quantifies three domains, including pain (5 items), stiffness (2 items), and physical function (17 items), each scored on a 0–4 Likert scale (higher scores indicate worse function). NP was evaluated via painDETECT questionnaire [12], a validated tool for detecting NP in KOA with score ranges from 0 to 38. Patients scoring 0–12 were considered unlikely to have NP (no NP), scores between 13 and 18 indicates ambiguous pain (suspected NP), and scores of 19–38 are considered likely to have NP (> 90% probability).

Based on the painDETECT questionnaire, we divided all cases into two categories, including patients with “simple nociceptive pain” and patients with “nociceptive pain combined with NP” (summarized in the following as “probable NP”) [13, 14]. Further classification within the simple nociceptive pain group was performed using WOMAC pain items [9, 15]. Patients reporting pain exclusively during weight-bearing activities (walking, stair climbing, and standing) were classified as having simple mechanical pain, while those experiencing both weight-bearing and resting pain (sitting and lying down) were categorized as mixed mechanical pain. Therefore, we summarized the pain types of KOA patients into three types, which are simple mechanical pain, mixed mechanical pain, and probable NP.

### 2.4. Statistical Analysis

Data analysis and statistics were processed using SPSS 20.0. Nonnormally distributed data were reported as the mean and 95% CI. Guided by the results of the Kolmogorov–Smirnov test, the Kruskal–Wallis test (with all pairwise comparisons Bonferroni corrected) and Mann–Whitney *U* tests were employed for intergroup comparisons of numerical values. Pearson's chi-square test was utilized to assess differences between categorical variables. Correlation analyses were performed using Spearman's rank correlation coefficients, with two-tailed *p* values serving as the basis for judgment. A *p* value less than 0.05 was considered indicative of statistically significant differences.

## 3. Results

### 3.1. Prevalence, Demographic Characteristics, and Knee Function Across the Three Groups

**Figure 1:**
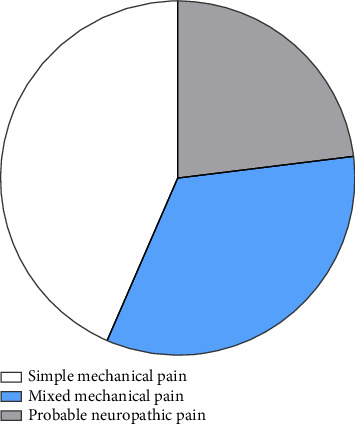
Prevalence of patients with three types of pain.

To evaluate the prevalence of mechanical pain in patients with KOA, we analyzed WOMAC pain scores and painDETECT questionnaire results from a cohort of 920 KOA patients. Our analysis revealed that 400 patients experienced simple mechanical pain, 308 exhibited mixed mechanical pain, and 212 reported probable NP. As illustrated in Figure 1, the prevalence rates were 43.48% for simple mechanical pain, 33.48% for mixed mechanical pain, and 23.04% for probable NP.

**Figure 2:**
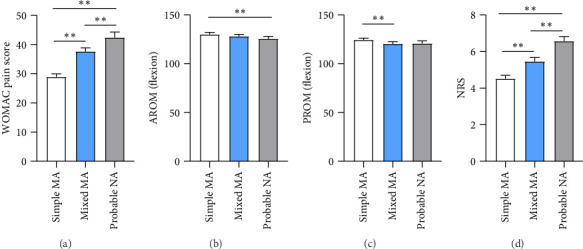
Comparison of knee parameters among patients with three types of pain. (a) WOMAC pain score. (b) Affected side-AROM (bend). (c) Affected side-PROM (bend). (d) Affected side-NRS. ^∗∗^*p* < 0.01. (Abbreviation: MA, mechanical pain; NA, neuropathic pain).

To investigate potential demographic and knee function differences, we compared the differences in age, BMI, WOMAC total score, range of motion, degree of varus, and NRS score among the three groups, as well as the WOMAC score between gender and the affected knee side using the Kruskal–Wallis test and Pearson's chi-square test. No significant differences were observed in age (*p* = 0.113), BMI (*p* = 0.661), or range of motion, including affected-side active range of motion (AROM) (straighten) (*p* = 0.126), passive range of motion (PROM) (straighten) (*p* = 0.054), or varus degree (*p* = 0.052). Additionally, no statistically significant differences were found in the total WOMAC scores for left and right knees across the groups. However, significant differences were identified in the total WOMAC scores, range of motion (bend), and NRS score among the three groups (Figure 2), significant differences in total WOMAC scores were also observed between male and female patients. Notably, gender differences were also significant among the three groups (^∗∗∗^*p* < 0.001). Specifically, the simple mechanical pain group consisted of 62.25% female patients, while the proportion of females increased to 75.97% in the mixed mechanical pain group and reached 80.66% in the probable NP group. As shown in Table 2, female patients outnumbered male patients across all pain types in each group. Additionally, it is noteworthy that pain manifestations were more prevalent on the right side compared with the left across all patient groups.

### 3.2. Risk Factors of Mechanical Pain

Given that mechanical pain exhibited the highest prevalence among the studied population, identifying its risk factors could assist clinicians in accurately diagnosing KOA patients with mechanical pain and formulating effective treatment strategies. Consequently, our analysis focused primarily on risk factors associated with simple mechanical pain and mixed mechanical pain. To investigate potential risk factors for mechanical pain, we employed Spearman's correlation and the Mann–Whitney *U* test to assess the relationships between age, sex, BMI, affected knee side, range of motion, degree of varus, and NRS. Our analysis revealed no significant correlation between age, BMI, range of motion, or degree of varus and the WOMAC pain score in patients with mechanical pain. However, the NRS on the affected side demonstrated a moderate correlation with the total WOMAC pain score (*r* = 0.500, ^∗∗∗^*p* < 0.001), as shown in Table 3. Thus, patients reporting higher NRS score were more likely to have worse mechanical pain-related dysfunction.

Additionally, we observed a significant sex-based difference in the WOMAC pain score (^∗^*p* < 0.05). Female patients exhibited a higher mean total WOMAC pain score (6.28) compared with male patients (6.08). Further analysis indicated that women with a WOMAC pain score > 4 had an odds ratio (OR) of 2.462 (95% CI: 1.766–3.433, ^∗^*p* < 0.05) relative to those with a score ≤ 4. These results suggested that female sex is an independent risk factor for greater functional impairment and mechanical pain severity in KOA. Given these results, we recommend prioritizing early assessment and targeted interventions for female KOA patients with mechanical pain, as this approach may facilitate timely pain relief and improve clinical outcomes.

## 4. Discussion

This study demonstrated that mechanical pain represents the predominant pain phenotype in advanced KOA, affecting 76.96% of the patients in our cohort. Notably, patients with high NRS score and female patients exhibited more severe knee damage and greater pain perception. Implementing targeted measures early on for this subset of patients would alleviate a significant portion of the challenges faced by those with KOA.

### 4.1. Prevalence and Treatment of Mechanical Pain

Mechanical pain is mediated by MSICs, including Piezo1, Piezo2, and TRPV4, which convert mechanical stimulation into electrochemical signals [16, 17]. Mechanical allodynia, a painful response to innocuous mechanical stimuli such as joint movement, is caused by the sensitization of articular nociceptors to mechanical stimuli [18]. Animal experiment has further demonstrated that nociceptors can be activated by mild mechanical stimulation, as evidenced by knee-bend tests [8]. Increased mechanical load, such as obesity, climbing, and weight-bearing exercises, is a critical factor in the progression of KOA, accelerating articular cartilage degeneration and activating MSICs across multiple joint structures, leading to persistent nociceptor activation and pain signal transmission to the brain via nerve fibers [19, 20]. In this study, patients with simple nociceptive pain were classified based on their WOMAC pain scores. Those experiencing pain exclusively triggered by mechanical joint loading during activities such as “walking, stair climbing, or standing” were categorized as having “simple mechanical pain.” In contrast, patients reporting pain even during rest, such as while “sitting or lying down,” were classified as having “mixed mechanical pain,” indicating the coexistence of mechanical pain with other pain types, such as inflammatory pain. The prevalence rates of simple mechanical pain and mixed mechanical pain were 45.8% and 33.48%, respectively. Notably, we did not find similar prevalence reports in the existing literature. Our analysis highlighted that mechanical pain constitutes a significant proportion of pain experienced by patients with KOA, offering valuable insights for tailoring treatment strategies for this population.

Effective management of mechanical pain in KOA requires a multimodal approach to relieve the mechanical stress and control the transduction of mechanical pain. Current therapeutic strategies primarily focus on weight management, targeted exercise regimens, pharmacological interventions, and surgical options when indicated. Reducing mechanical stress stimulation is an important part of reducing mechanical pain, such as weight control, decreasing the load, weight-free exercise (swimming, cycling, etc.), and strengthening the muscles around the knee joint. A clinical study showed that ultrasound and extracorporeal shock wave therapy (ESWT) statistically reduced pain and disability and combined with other therapies significantly improved resting pain, WOMAC outcomes, and physical function. Notably, the combination of vibratory stimulation with conventional exercise protocols appears superior to exercise alone, particularly in improving pain intensity and WOMAC outcomes [21]. For patients with advanced structural damage, surgical interventions such as periknee osteotomy offer both pain relief and functional preservation [22]. These procedures maintain native joint anatomy while addressing mechanical contributors to pain generation. Moreover, Identifying key targets within mechanosensitive pathways that contribute to mechanical pain in KOA could unveil novel therapeutic strategies for treating KOA following cartilage injury or trauma. For instance, compounds like GsMTx4, anti-nerve growth factor (NGF) monoclonal antibodies, and verapamil, which target Piezo channels and L-type voltage-gated Ca2+ channels, respectively, present promising options for the development of targeted therapies for mechanical pain management [17, 23].

### 4.2. Risk Factors of Mechanical Pain in Patients With KOA

Previous studies have established multiple risk factors for OA development, including old age, femininity, obesity, knee injury, repeated joint use, bone density, muscle weakness, and joint laxity [24]. This study showed significant differences in gender, total WOMAC scores, range of motion (bend), and NRS score among the three groups of patients with simple mechanical pain, mixed mechanical pain, and probable NP. The observed sex-based differences in clinical manifestations may be attributed to several biological factors. Anatomical variations, distinct kinematic patterns, differential knee injury prevalence, and hormonal influences collectively contribute to increased KOA susceptibility and greater pain severity in female patients [25, 26]. This study showed that female patients exhibited significantly higher WOMAC pain score compared with males and particularly highlighted that women with simple mechanical pain exhibited significantly higher WOMAC pain score than male counterparts, with an odds ratio of 2.462. This finding aligns with previous reports indicating more severe radiographic changes and symptom burden in female OA patients [27]. These results suggested that female sex is an independent risk factor for greater functional impairment and mechanical pain severity in KOA, and female patients may experience more complex pain phenotypes and greater pain intensity. Thus, taking targeted measures in advance and regularly screening these patients may prevent or delay the occurrence of pain. The observed total WOMAC scores differences across groups reflect our classification methodology. The simple mechanical pain group demonstrated the lowest WOMAC scores, representing a single pain mechanism. Conversely, the probable NP group displayed the highest WOMAC scores, which may be attributed to the similar trend observed between WOMAC scores and NP severity, which is significantly correlated with painDETECT scores [29]. Consequently, higher NP scores naturally correspond to elevated WOMAC scores. There were significant differences in the range of motion of the bent knee in the three groups but no significant differences in straightened knee, which may be related to the bias in the range of motion measurement and the limited data included. In this study, NRS score varied significantly among groups, with the mechanical pain group showing the lowest values and the probable NP group the highest, which was consistent with our classification approach. Notably, the moderate correlation between NRS score and WOMAC pain score suggested that NRS may serve as a useful clinical indicator for identifying patients at risk of significant mechanical pain-related disability. These findings underscore the need for comprehensive risk factor assessment in mechanical pain of KOA management. Future research should explore additional predictive factors to enhance clinical identification of patients at risk for mechanical pain.

## 5. Limitations

Currently, standardized classification criteria for mechanical pain remain undefined in the literature, and existing classification methods necessitate further experimental validation. In this study, mechanical pain was classified based on the WOMAC scores and NP assessment, which provides a reasonable degree of representativeness. This classification approach will be adopted in subsequent research. Additionally, our analysis included a limited set of risk factors, such as age, sex, BMI, affected side of knee, range of motion, degree of varus, and NRS score. However, the potential risk factors, including knee swelling and the concentration of inflammatory mediators in synovial fluid [28, 29], may significantly influence mechanical pain. Therefore, future investigations should aim to incorporate these additional variables to more comprehensively explore the full spectrum of risk factors contributing to mechanical pain.

## 6. Conclusions

In conclusion, this study showed that patients with mechanical pain accounted for a large proportion of patients with KOA, and female or higher NRS score of KOA patients were more likely to have mechanical pain. These findings could help guide the future treatment of mechanical pain in patients with KOA and help doctors take some preventive and targeted measures to provide personalized treatment strategies for patients with mechanical pain. We suggest that future clinical trials may be conducted to clarify the measurement criteria and treatment methods for mechanical pain.

## Figures and Tables

**Table 1:** Patient demographics and affected knee parameters.

	Value
Age (year)	69.93 (69.47–70.41)
Male/Female	273/663
BMI (kg/m^2^)	25.87 (25.64–26.10)
Left/right side	410/526
WOMAC	36.43 (35.61–37.25)
Range of motion (degree)	
AROM: straighten	8.50 (7.93–9.07)
AROM: bend	124.18 (123.04–125.31)
PROM: straighten	9.13 (8.58–9.68)
PROM: bend	122.37 (121.19–123.55)
Degree of varus (degree)	8.68 (8.40–8.95)
NRS	5.25 (5.12–5.39)

*Note:* All knee parameters were evaluated on affected sides, including WOMAC, range of motion, degree of varus, and NRS, and this protocol applies to all subsequent data presentations in this study. The values in parentheses represent the mean and 95% CI, a format consistently maintained throughout the tables. “Degree of varus” includes both varus and valgus, with valgus deformities represented as absolute values. This convention applies to all angular measurements reported in this study.

Abbreviations: BMI, body mass index; WOMAC, Western Ontario and McMaster Universities Osteoarthritis Index; AROM, active range of motion; PROM, passive range of motion; NRS, numeric rating scale.

**Table 2:** Characteristics of patients with three types of pain.

	Simple mechanical pain (*n* = 400)	Mixed mechanical pain (*n* = 308)	Probable neuropathic pain (*n* = 212)
Age (year)	70.54 (69.80–71.27)	69.36 (68.58–70.15)	69.90 (68.85–70.95)
Male/Female	151/249	74/234	41/171
BMI (kg/m^2^)	25.69	26.06	26
Left/Right side	177/223	125/182	100/112
WOMAC	30.72 (29.74–31.70)	39.23 (37.97–40.49)	44.47 (42.68–49.26)
Range of motion (degree)			
AROM: straighten	8.18 (7.28–9.09)	8.53 (7.57–9.50)	9.29 (8.10–10.48)
AROM: bend	126.03 (124.40–127.65)	123.56 (121.59–125.53)	121.62 (118.99–124.25)
PROM: straighten	8.43 (7.63–9.23)	9.78 (8.90–10.66)	9.66 (8.31–11.01)
PROM: bend	124.63 (123.00–126.24)	120.48 (118.37–122.59)	120.91 (118.18–123.63)
Degree of varus (degree)	8.55 (8.12–8.98)	9.03 (8.57–9.50)	8.30 (7.73–8.87)
NRS	4.53 (4.35–4.72)	5.46 (5.24–5.67)	6.57 (6.32–6.83)

**Table 3:** Correlation between variables and total WOMAC pain score.

	Correlation (*r*)	Sig (*p*)
Age (year)	−0.020	0.598
BMI (kg/m^2^)	0.051	0.176
Range of motion (degree)		
AROM: straighten	0.088	0.019^∗^
AROM: bend	−0.099	0.008^∗∗^
PROM: straighten	0.137	< 0.001^∗∗∗^
PROM: bend	−0.115	0.002^∗∗^
Degree of varus (degree)	0.133	< 0.001^∗∗∗^
NRS	0.500	< 0.001^∗∗∗^

^∗^ *p* < 0.05.

^∗∗^ *p* < 0.01.

^∗∗∗^ *p* < 0.001.

## Data Availability

Clinical data are subject to privacy restrictions and available on reasonable request to the corresponding author.

## Supporting Information

The graphical abstract illustrates mechanical stress induced pain in KOA patients. Our analysis of 920 patients revealed that 43.48% (400 patients) experienced simple mechanical pain, 33.48% (308 patients) exhibited mixed mechanical pain, and 23.04% (212 patients) reported probable neuropathic pain. Mechanical pain, typically induced by mechanical stress such as walking, stair climbing, or standing, was the most common type of pain in patients with advanced KOA.

## References

[1] Katz J. N., Arant K. R., Loeser R. F. (2021). Diagnosis and Treatment of Hip and Knee Osteoarthritis: A Review. *JAMA*.

[2] Michael J. W. P., Schlüter-Brust K. U., Eysel P. (2010). The Epidemiology, Etiology, Diagnosis, and Treatment of Osteoarthritis of the Knee. *Deutsches Arzteblatt International*.

[3] Zhang Y., Jordan J. M. (2010). Epidemiology of Osteoarthritis. *Clinics in Geriatric Medicine*.

[4] Vina E. R., Kwoh C. K. (2018). Epidemiology of Osteoarthritis: Literature Update. *Current Opinion in Rheumatology*.

[5] Kosek E., Cohen M., Baron R. (2016). Do We Need a Third Mechanistic Descriptor for Chronic Pain States?. *Pain*.

[6] Nijs J., Lahousse A., Kapreli E. (2021). Nociplastic Pain Criteria or Recognition of Central Sensitization? Pain Phenotyping in the Past, Present and Future. *Journal of Clinical Medicine*.

[7] Fernández-De-Las-Peñas C., Nijs J., Neblett R. (2022). Phenotyping Post-COVID Pain as a Nociceptive, Neuropathic, or Nociplastic Pain Condition. *Biomedicines*.

[8] He B. H., Christin M., Mouchbahani-Constance S., Davidova A., Sharif-Naeini R. (2017). Mechanosensitive Ion Channels in Articular Nociceptors Drive Mechanical Allodynia in Osteoarthritis. *Osteoarthritis and Cartilage*.

[9] Qiyao L., Chengchang H., Rui H., Xiang G., Li L., Pei F. (2024). Research Progress on Mechanical Pain in Knee Osteoarthritis. *Chinese Journal of Bone and Joint Surgery*.

[10] Zhang M., Wang Y., Geng J., Zhou S., Xiao B. (2019). Mechanically Activated Piezo Channels Mediate Touch and Suppress Acute Mechanical Pain Response in Mice. *Cell Reports*.

[11] MacKay C., Clements N., Wong R., Davis A. M. (2019). A Systematic Review of Estimates of the Minimal Clinically Important Difference and Patient Acceptable Symptom State of the Western Ontario and Mcmaster Universities Osteoarthritis Index in Patients Who Underwent Total Hip and Total Knee Replacement. *Osteoarthritis and Cartilage*.

[12] Freynhagen R., Baron R., Gockel U., Tölle T. R. (2006). Paindetect: A New Screening Questionnaire to Identify Neuropathic Components in Patients With Back Pain. *Current Medical Research and Opinion*.

[13] Treede R. D., Jensen T. S., Campbell J. N. (2008). Neuropathic Pain: Redefinition and a Grading System for Clinical and Research Purposes. *Neurology*.

[14] Freynhagen R., Tölle T. R., Gockel U., Baron R. (2016). The Paindetect Project-Far More Than a Screening Tool on Neuropathic Pain. *Current Medical Research and Opinion*.

[15] Woolacott N. F., Corbett M. S., Rice S. J. (2012). The Use and Reporting of WomaC in the Assessment of the Benefit of Physical Therapies for the Pain of Osteoarthritis of the Knee: Findings From a Systematic Review of Clinical Trials. *Rheumatology*.

[16] Nims R., Palmer D. R., Kassab J., Zhang B., Guilak F. (2024). The Chondrocyte “Mechanome”: Activation of the Mechanosensitive Ion Channels Trpv4 and PiezO1 Drives Unique Transcriptional Signatures. *The FaseB Journal*.

[17] Gao W., Hasan H., Anderson D. E., Lee W. (2022). The Role of Mechanically-Activated Ion Channels Piezo1, Piezo2, and Trpv4 in Chondrocyte Mechanotransduction and Mechano-Therapeutics for Osteoarthritis. *Frontiers in Cell and Developmental Biology*.

[18] Sliwinski C., Heutehaus L., Taberner F. J. (2024). Contribution of Mechanoreceptors to Spinal Cord Injury-Induced Mechanical Allodynia. *Pain*.

[19] Vincent T. L. (2019). Mechanoflammation in Osteoarthritis Pathogenesis. *Seminars in Arthritis and Rheumatism*.

[20] Zeng C. Y., Zhang Z. R., Tang Z. M., Hua F. Z. (2021). Benefits and Mechanisms of Exercise Training for Knee Osteoarthritis. *Frontiers in Physiology*.

[21] Oliveira S., Andrade R., Valente C. (2022). Mechanical-Based Therapies May Reduce Pain and Disability in Some Patients With Knee Osteoarthritis: A Systematic Review With Meta-Analysis. *The Knee*.

[22] Ogawa H., Matsumoto K., Ogawa T., Takeuchi K., Akiyama H. (2016). Preoperative Varus Laxity Correlates With Overcorrection in Medial Opening Wedge High Tibial Osteotomy. *Archives of Orthopaedic and Trauma Surgery*.

[23] Obeidat A. M., Wood M. J., Adamczyk N. S. (2023). Piezo2 Expressing Nociceptors Mediate Mechanical Sensitization in Experimental Osteoarthritis. *Nature Communications*.

[24] Martel-Pelletier J., Barr A. J., Cicuttini F. M. (2016). Osteoarthritis. *Nature Reviews Disease Primers*.

[25] Hame S. L., Alexander R. A. (2013). Knee Osteoarthritis in Women. *Current Reviews In Musculoskeletal Medicine*.

[26] Long H., Zeng X., Liu Q. (2020). Burden of Osteoarthritis in China, 1990-2017: Findings From the Global Burden of Disease Study 2017. *The Lancet. Rheumatology*.

[27] Srikanth V. K., Fryer J. L., Zhai G., Winzenberg T. M., Hosmer D., Jones G. (2005). A Meta-Analysis of Sex Differences Prevalence, Incidence and Severity of Osteoarthritis. *Osteoarthritis and Cartilage*.

[28] Jang S., Lee K., Ju J. H. (2021). Recent Updates of Diagnosis, Pathophysiology, and Treatment on Osteoarthritis of the Knee. *International Journal of Molecular Sciences*.

[29] Mathiessen A., Conaghan P. G. (2017). Synovitis in Osteoarthritis: Current Understanding With Therapeutic Implications. *Arthritis Research and Therapy*.

