# Supplementary material for: Mechanical Pain is a Main Type of Pain in Patients With Advanced Knee Osteoarthritis
Source: Pain Res Manag. 2025 Nov 24;2025:8356050. doi: 10.1155/prm/8356050 (PMC12668844; doi:10.1155/prm/8356050)

Mechanical stress induced pain in knee osteoarthritis patients

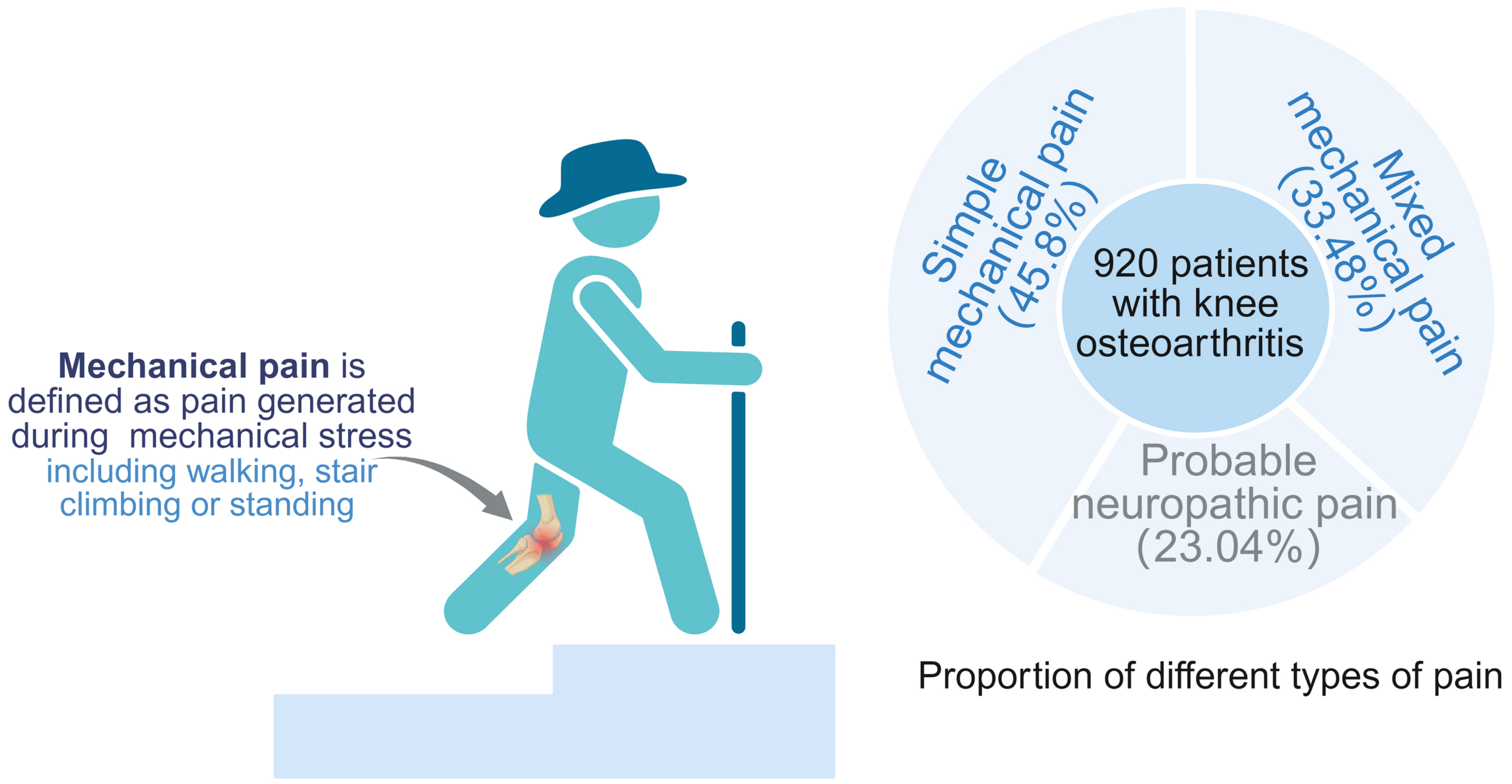

Supplement: Supporting Information — Additional supporting information can be found online in the Supporting Information section. [file 8356050.f1.pdf]
